# Infrared thermography as an access pathway for individuals with severe motor impairments

**DOI:** 10.1186/1743-0003-6-11

**Published:** 2009-04-16

**Authors:** Negar Memarian, Anastasios N Venetsanopoulos, Tom Chau

**Affiliations:** 1 Institute of Biomaterials and Biomedical Engineering, University of Toronto, Toronto, Canada; 2 Bloorview Research Institute, Bloorview Kids Rehab, Toronto, Canada; 3 Department of Electrical and Computer Engineering, University of Toronto, Toronto, Canada; 4 Department of Electrical and Computer Engineering, Ryerson University, Toronto, Canada

## Abstract

**Background:**

People with severe motor impairments often require an alternative access pathway, such as a binary switch, to communicate and to interact with their environment. A wide range of access pathways have been developed from simple mechanical switches to sophisticated physiological ones. In this manuscript we report the inaugural investigation of infrared thermography as a non-invasive and non-contact access pathway by which individuals with disabilities can interact and perhaps eventually communicate.

**Methods:**

Our method exploits the local temperature changes associated with mouth opening/closing to enable a highly sensitive and specific binary switch. Ten participants (two with severe disabilities) provided examples of mouth opening and closing. Thermographic videos of each participant were recorded with an infrared thermal camera and processed using a computerized algorithm. The algorithm detected a mouth open-close pattern using a combination of adaptive thermal intensity filtering, motion tracking and morphological analysis.

**Results:**

High detection sensitivity and low error rate were achieved for the majority of the participants (mean sensitivity of all participants: 88.5% ± 11.3; mean specificity of all participants: 99.4% ± 0.7). The algorithm performance was robust against participant motion and changes in the background scene.

**Conclusion:**

Our findings suggest that further research on the infrared thermographic access pathway is warranted. Flexible camera location, convenience of use and robustness to ambient lighting levels, changes in background scene and extraneous body movements make this a potential new access modality that can be used night or day in unconstrained environments.

## Background

### Alternative access pathways

Individuals with severe physical impairments who are unable to communicate through speech or gestures require an alternative means to convey their intentions. In the rehabilitation engineering context, these alternative channels are called access pathways and they constitute the critical front end of an access solution [1]. Some recent efforts have set out to non-invasively translate physiological signals such as the electrical [2,3] and hemodynamic activity [4-6] of the brain or the electrodermal response of the skin [7,8] into functional communication. A comprehensive review of emerging access technologies can be found in [1].

### Biomedical applications of thermal imaging

Infrared thermography refers to the measurement of the radiation emitted by the surface of an object in the infrared range of the electromagnetic spectrum, i.e., between wavelengths of 0.8 μm and 1.0 mm [9]. Infrared cameras use specialized lenses manufactured from materials such as germanium to focus thermal radiation onto a focal plane array of infrared detectors [10]. Thermal cameras yield an image that is a spatial, two-dimensional (2-D) map of the 3-D temperature distribution of the object [11].

Infrared thermography has been widely applied in health research, including, for example, breast cancer detection [12,13], brain surgery [14,15], heart surgery [16], diagnosis of vascular disorders [17], arthritis [18], pain assessment [19] and post-surgical follow-up in ophthalmology [20].

Recently, Murthy and Pavlidis non-invasively measured human breathing using infrared imaging and a statistical methodology based on multinormal distributions, the method of moments, and Jeffreys divergence measure [21]. Their study was based on the fact that exhaled gases have a higher temperature than the typical background of indoor environments. They achieved high detection accuracy on a small set of subjects and suggested potential applications in polygraphy, sleep studies, sport training, and patient monitoring [21].

### Thermal imaging as an access pathway

The goal of this paper is to investigate the potential of thermal imaging as an access pathway. In particular, we introduce a thermographic binary switch activated by voluntary mouth opening. Expired air and the oral cavity are generally warmer than the surrounding tissue and environment while cyclic jaw movements do not cause significant increases in facial temperatures over time [22]. Therefore localized temperature changes due to mouth opening and closing may be detectable using video and image processing of thermographic data. Examples of patient groups that may benefit from this access pathway are people with high level spinal cord injuries resulting in quadriplegia and individuals with spastic quadriplegic cerebral palsy or general hypotonia.

Like computer vision-based access pathways [23], thermal imaging is non-invasive and does not require any sensor attachment to the user. However, thermography overcomes some of the major limitations of conventional computer vision-based access pathways. Firstly, thermography is skin colour invariant since there is no difference in emissivity between black, white and burnt skin, in vivo or in vitro [24]. Human skin has an emissivity of about 0.98. Thermal radiation from the skin originates in the epidermis and is independent of race; it depends therefore only on the surface temperature [9,11]. Secondly, thermal image quality is independent of ambient lighting conditions and can thus be effective both night and day. Conceivably, this non-contact, non-invasive access pathway could be tailored to the user's unique motor capacity, whether that be mouth opening, eye blinking or simply deep breathing. These are all motor activities that may generate measurable, local temperature changes. Furthermore, given that the key information is thermal variation, a frontal view of the user may not be necessary, facilitating more flexible and unobtrusive placement of the camera.

## Methods

### Participants

Eight able-bodied participants and two individuals with quadriplegia (one with a C1-C2 incomplete spinal cord injury and the other with severe spastic quadriplegic cerebral palsy) participated in this study. All participants provided written consent. The experimental protocol was approved by the research ethics board of the university and affiliated hospital.

### Instrumentation and setup

A THERMAL-EYE 2000B thermal video camera by L-3 Communications with thermal sensitivity ≤100 *mK *[25] was connected via an NTSC to USB TV convertor (Dazzle Multimedia). Videos were recorded as 240 × 320 AVI files (30 fps) and processed offline in MATLAB & Simulink (version R2007b).

Participants were comfortably seated within a laboratory environment. Those with disability remained in their wheelchairs. The thermal camera was positioned anterior and lateral to the participant at a 45° angle. This camera location was chosen over the often-used frontal view, keeping in mind the eventual application as an access switch where the user's field of view ought to be unobstructed. In the 45° angle condition, infrared thermograms only exhibit a small error in recorded temperatures [9]. Each participant was cued to open his or her mouth and to hold it ajar for one second before closing the mouth. Participants were given an auditory prompt upon every open and close action. The end of each mouth closing was followed by a 3 second rest before the onset of the next mouth opening. The participants were instructed to maintain a constant head position, so that their mouth movement stayed within the camera's field of view.

The thermal sensitivity of the infrared camera we used was well beyond what was needed to detect the temperature change due to mouth opening. We are looking at temperature difference of about 1.5 to 3°C between when mouth is closed and when it is open, while the thermal sensitivity of our infrared camera was ≤100 *mK*.

### Thermal video processing

**Figure 1:**
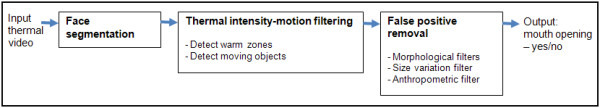
**Components of the proposed mouth opening detection algorithm**.

Figure 1 shows a schematic of our algorithm for detecting mouth openings from the thermal video data. The system consisted of three main components, namely face segmentation, thermal intensity-motion filtering and false positive removal. Each component will be discussed below. To begin, the boundary pixels of each video frame (the first and last pixels of every column and every row) were set to zero to detach objects that may be connected to the borders.

#### Face segmentation

In addition to the participant's head and facial region, other body parts such as the participant's neck, thorax and upper limbs also appeared in the videos. For the participants with disability, parts of their wheelchairs were also captured on thermal video. Objects in the background, and in a couple of instances people moving around the participant were also recorded. It was thus essential to segment the participant's face region from all other non-target body parts and objects. Each frame of the video was binarized. Given that facial temperature distributions vary within and among individuals [26], we adopted Otsu's method to determine an adaptive rather than fixed intensity threshold which minimized, on a frame by frame basis, the intra-class variance of the grayscale values of the pixels to be binarized [27].

**Figure 2:**
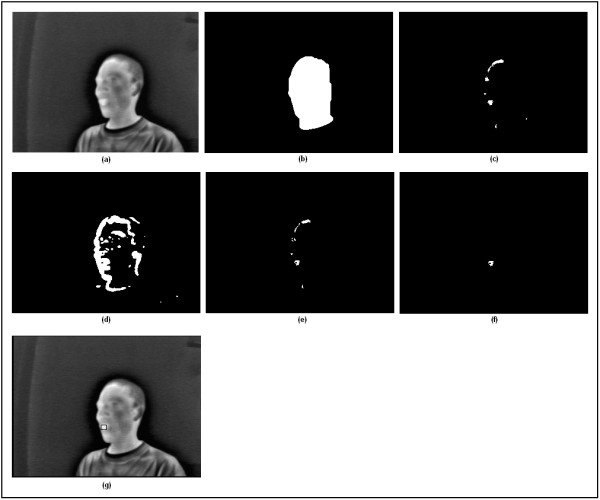
**The action of the different modules of the mouth opening detection algorithm**. (a) Input thermal video frame, (b) Segmented face region, (c) Warm facial zones, (d) Moving facial zones, (e) Intersection of warm and moving objects within the face region, (f) After morphological, size variation, and anthropometric filtering, (g) Final output; detected mouth open is highlighted on the original video with a hollow box.

The binarized frames were then morphologically opened with a disk structuring element of radius 5 pixels to remove small objects, break thin connections, remove thin protrusions, and smooth object contours [28]. In the resulting image, the object with maximum area (presumably the face region) was retained and the object's interior holes were filled by morphological closing with a disk structuring element of radius 20 pixels. The camera-user distance and the user's head size affect the dimension of the above mentioned structuring elements. In a real life application, the camera will be mounted on the user's wheelchair at a fixed distance from the user's face. Hence, once the appropriate parameters are selected in the initial calibration, they do not need to be changed for subsequent use. An example of a segmented face region is depicted in Figure 2(b).

#### Thermal intensity-motion filtering

All subsequent processing was applied to the intensity image and confined to the identified face region. The region of interest (ROI) was the participant's mouth and the task of interest was mouth opening. A combination of temperature thresholding and motion tracking was used to perceive mouth opening. Warm zones inside the facial region were extracted by thresholding the segmented face with a scaled version of Otsu's threshold [27] to favour higher intensity (i.e., warmer) pixels. The scale factor was empirically derived as

(1)

and typically ranged from 2.5 to 3. This segmentation yielded a warm zone mask which served to detect instances of mouth opening. However, there were occasions where nearby facial regions had similar temperatures as those of the oral cavity. A corroborating cue was therefore required to accurately pinpoint a mouth opening event.

Since mouth opening involves motion, optical flow was utilized to estimate the direction and speed of motion from one video frame to the next using the Horn-Schunck method [29]. Motion vectors in each frame of the video sequence were computed by solving the optical flow constraint equation

(2)

where *I*_*x*_, *I*_*y *_and *I*_*t *_are the spatiotemporal image brightness derivatives, *u *is the horizontal optical flow and *v *is the vertical optical flow. By assuming that the optical flow is smooth over the entire image, the Horn-Schunck method computes an estimate of the velocity field, [*u v *]^*T*^, that minimizes this equation:

(3)

In this equation  and  are the spatial derivatives of the optical velocity component *u*, and *α *scales the global smoothness term [29]. Motion vectors with velocity magnitude exceeding the mean velocity (i.e., the average of velocity magnitudes across the most recent five frames) per frame across time were retained, yielding a motion mask. The intersection of this motion mask and the warm zone mask, introduced above, yielded all the regions of the face that were both warm and moving.

#### False positive removal

Despite the combination of motion and thermal cues, the processed frames occasionally contained non-mouth objects (false positives) such as parts of the chin, forehead and the periorbital regions. These non-mouth objects were also warm and moving and were therefore retained subsequent to the thermal intensity and motion filters. An example is the forehead, which according to the literature, is the warmest part of the human body with a temperature (34.5°C) close to that inside the mouth [30]. Therefore motion of the forehead may result in a false positive.

To deal with these false positives, we deployed a series of additional filters based on morphology, size variation between frames, and facial anthropometry. Objects that did not meet the following morphological conditions were deemed as false positives and removed.

1. 30 pixels < Area < 150 pixels

2. Eccentricity ≤ 0.9.

3. 

The first condition rejects objects which are either too small or too large to be candidate mouth openings. Likewise, the second condition removes regions that are too elongated to qualify as mouth regions while the third condition eliminates hollow regions as the mouth is expected to be solid. The constants in these morphological filters were selected to resemble the shape of the open mouth and were empirically defined. In addition, objects whose size varied less than 25% between the current frame and the frame occurring ten frames earlier were considered static warm facial regions (e.g., forehead, chin, around the eyes, neck) and were also discarded. This constitutes the size variation filter in Figure 1.

Finally we exploited the fact that facial anatomy is static (i.e., unlikely to change over time). Based on human face anthropometry, the mouth is located in the lower half of the menton-sellion length [31,32]. When we partitioned the facial ROI along its major axis into four strips, we noticed that indeed the mouth was usually located in the second strip from the bottom. With this anthropometric filter, we dismissed candidate ROIs outside of the second facial quarter. Figures 2(c)–(g) demonstrate the action of the different processing modules.

### Algorithm evaluation

To facilitate algorithm evaluation, a truth set was prepared manually for each recorded thermal video. The truth set contained the frame numbers corresponding to the beginning and ending of each mouth opening, the end points of the line maximally spanning the width of the mouth at the onset of opening and the end points of the line maximally spanning the height of the mouth when fully ajar. This truth set served as the gold standard for automatic algorithm evaluation. A true positive was defined as the detection of a ROI temporally within the range of frames corresponding to a gold standard mouth opening, and spatially situated within the bounding box defined by the endpoints extracted above. All other detected objects were considered false positives. A mouth opening that was missed by the algorithm was counted as a false negative. A true negative occurred when there was no mouth opening and the algorithm concluded the same. Sensitivity and specificity values were estimated.

## Results and discussion

**Figure 3:**
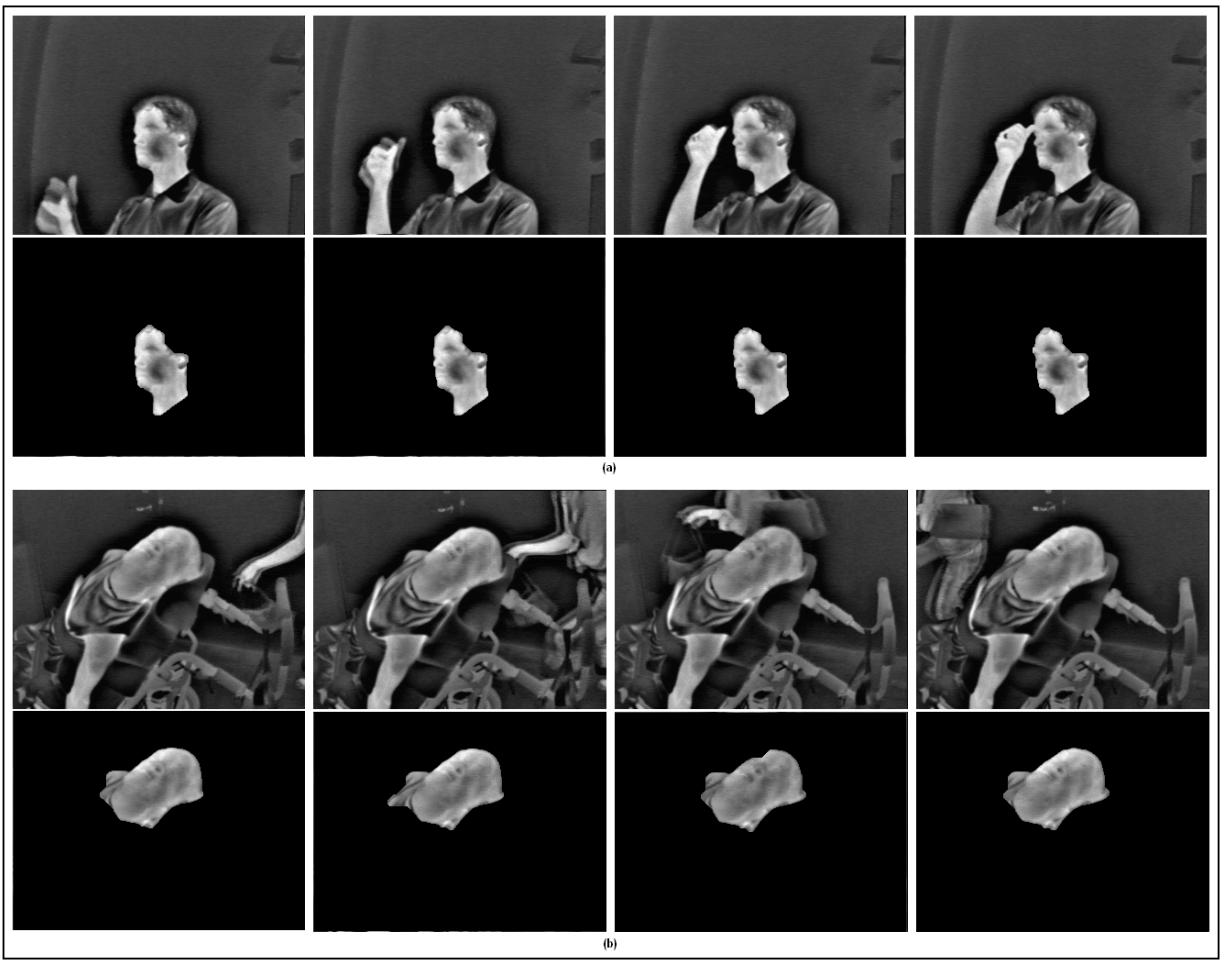
**Robustness of the proposed algorithm to motion artefacts and changes in the background**. (a) Robustness to motion artefacts. Top row from left to right shows input thermal video of an able-bodied participant moving his arm to his head (frames 63, 66, 70, and 74). Bottom row depicts face segmentation in the corresponding frames. (b) Robustness to changes in the background. Top row from left to right is an input thermal video of a participant with disability while a passerby traverses the scene in the background (frames 1759, 1765, 1779, 1790). The corresponding face segmentation results are presented in the bottom row.

The performance of the proposed algorithm on the thermal video of ten participants is summarized in Table 1. Detection of mouth opening is generally achieved with very high sensitivity and specificity. The exception is the poorer result for participant 10, which is mainly due to participant's posture, frequent involuntary head rotation away from the camera, and suboptimal camera placement. This participant had an awkward position in his wheelchair (See Figure 3(b)) which forced us to position the thermal camera at an angle and distance from the participant that was not consistent with the other participants. Several improvements can be made to enhance the results in situations like this: (1) The algorithm can be updated to track and focus on the region of interest (participant's face) more accurately; (2) Multiple cameras can be used to capture participant's facial region from different angles, so that the problem of participant mouth leaving the camera's field of view will be mitigated; and (3) The user can be trained. Figures reported in the present paper are the result of just one test session. Training is expected to have a positive effect on user performance.

Specificity is generally higher than sensitivity as the algorithm was tuned to minimize false positives, again keeping in mind the alternative access application where inadvertent switch activations are arguably more costly than missed activations. Most of the false positives were repeated detections of the same non-mouth object in multiple frames. The chin was the source of the majority of the false positives, which tended to occur during actual mouth openings. This is perhaps not surprising given that the chin is proximal to the mouth and moves as the jaw descends to open the mouth. Further, the chin is reportedly the warmest facial area after the forehead [33] when measured by thermography.

The proposed algorithm is robust against participant motion and changes to the background scene. Figure 3(a) demonstrates an example of one of the participants moving his arm towards his face. Although the arm is both warm and moving, and even touches the participant's face in some frames, it was correctly disregarded by the algorithm. Figure 3(b) depicts an example of a person entering and leaving the background scene. The algorithm successfully rejected the background activity and did not generate any false positives.

The proposed combination of filters is location and position invariant; regardless of where in the frame the user moves his or her head within the camera's field of view and independent of the user's position (sitting or semi-supine), mouth opening could generally be located relative to the segmented face region.

**Table 1:** Performance of the proposed mouth opening detection algorithm

**Participant**	**Video length (sec)**	**Total Video frames**	**Actual # of mouth openings**	**Sensitivity**	**Specificity**
1	256	7662	50	88%	100%
2	252	7546	50	96%	100%
3	254	7621	50	96%	100%
4	252	7481	50	98%	100%
5	244	7424	50	88%	99%
6	243	7594	50	92%	98%
7	245	7664	50	94%	99%
8	243	7613	50	80%	100%
9*	153	4592	30	93%	99%
10*	272	8160	15	60%	99%

*Participant with severe disability.

If one can voluntary control mouth open and close action, sip and puff technology, EMG based switches, and computer vision based switches can also be used. The advantage of the proposed thermography based access pathway over sip and puff and EMG based switches is that it is non-invasive and non-contact, i.e., does not require attachment of any sensor or external object to the user. Hence it is more hygienic and safe, as the risk of choking is also eliminated. Its advantage over visible light computer vision based access pathways is that it is independent of lighting/color and can thus be used both night and day, indoor and outdoor.

Despite these encouraging findings, thermal imaging does have its limitations. Infrared thermal cameras are more expensive than conventional (visible light) cameras. However, recent innovations in affordable, pocket sized, portable thermal cameras [34] may eventually eliminate the cost issue. Thermal image quality is susceptible to fluctuations in ambient temperature, humidity and regional air circulation [9]. A robust thermographic access pathway may need to dynamically compensate for changes in these contextual factors. A final limitation of thermal imaging is the relatively low resolution of infrared cameras and the inherent difficulty in discriminating between fine facial features. These issues may be mitigated by fusing thermal videos with simultaneously recorded visible spectrum imagery [35].

## Conclusion

We have demonstrated that infrared thermography can be used as a non-contact and non-invasive access pathway for individuals who retain voluntary mouth opening and closing. Our analyses suggest that the thermographic access pathway may be robust to various lighting levels, different body postures, extraneous user movements, and background variations.

## Competing interests

The authors declare that they have no competing interests.

## Authors' contributions

NM designed and implemented the video processing algorithm, performed the thermographic data analysis, and drafted the manuscript. ANV read the manuscript and commented on the methods. TC conceived the study and edited the manuscript. All authors read and approved the final manuscript.

## References

[B1] Tai K, Blain S, Chau T (2008). A review of emerging access technologies for individuals with severe motor impairments. Assistive Technology.

[B2] Sellers EW, Kubler A, Donchin E (2006). Brain-computer interface research at the university of South Florida cognitive psychophysiology laboratory: the P300 speller. IEEE Transactions on Neurological Systems and Rehabilitation Engineering (Special Issue on Brain-Computer Inerfaces).

[B3] Piccione F, Giorgi F, Tonin P, Priftis K, Giove S, Silvoni S, Palmas G, Beverina F (2006). P300-based brain computer interface: Reliability and performance in healthy and paralyzed participants. Clinical Neurophysiology.

[B4] Coyle SM, Ward TE, Markham CM (2007). Brain-computer interface using a simplified functional near-infrared spectroscopy system. J Neural Eng.

[B5] Sitaram R, Zhang H, Guan C, Thulasidas M, Hoshi Y, Ishikawa A, Shimizu K, Birbaumer N (2007). Temporal classification of multichannel near-infrared spectroscopy signals of motor imagery for developing a brain-computer interface. NeuroImage.

[B6] Naito M, Michioka Y, Ozawa K, Ito Y, Kiguchi M, Kanazawa T (2007). A communication means for totally locked-in ALS patients based on changes in cerebral blood volume measured with near-infrared light. IEICE Transactions on Information and Systems, E90D(7).

[B7] Blain S, Mihailidis A, Chau T (2008). Assessing the potential of electrodermal activity as an alternative access pathway. Medical engineering & physics.

[B8] Tsukahara R, Aoki H (2002). Skin potential response in letter recognition task as an alternative communication channel for individuals with severe motor disability. Clinical Neurophysiology.

[B9] Jones BF (1998). A reappraisal of the use of infrared thermal image analysis in medicine. IEEE Transactions on Medical Imaging.

[B10] Lupo J, Balcerak R (2000). The physical basis of thermal imaging. Proc 22nd Annual Conf IEEE Engineering in Medicine and Biology Society Chicago.

[B11] Jones BF, Plassmann P (2002). Digital infrared thermal imaging of human skin. IEEE Eng Med Biol Mag.

[B12] Qi H, Diakides NA (2003). Thermal Infrared Imaging in Early Breast Cancer Detection – A Survey of Recent Research. Proceedings of the 25' Annual International Conference of the IEEE EMBS Cancun, Mexico.

[B13] Gautherie M (1989). Atlas of breast themogmphy with specific guidelines for eramination and interpretation.

[B14] Okada Y, Kawamata T, Kawashima A, Hori T (2007). Intraoperative application of thermography in extracranial-intracranial bypass surgery. Neurosurgery.

[B15] Watson JC, Gorbach AM, Pluta RM, Rak R, Heiss JD, Oldfield EH (2002). Real-time detection of vascular occlusion and reperfusion of the brain during surgery by using infrared imaging. J Neurosurg.

[B16] Madjid M, Willerson JT, Casscells SW (2006). Intracoronary thermography for detection of high-risk vulnerable plaques. J Am Coll Cardiol.

[B17] Ring F, Harding R (2000). Infrared thermal imaging in peripheral vascular diseases. World Congress on Medical Physics and Biomedical Engineering Chicago.

[B18] Spalding SJ, Kwoh CK, Boudreau R, Enama J, Lunich J, Huber D, Denes L, Hirsch R (2008). Three-dimensional and thermal surface imaging produces reliable measures of joint shape and temperature: a potential tool for quantifying arthritis. Arthritis Research & Therapy.

[B19] Herry CL, Frize M (2004). Quantitative assessment of pain-related thermal dysfunction through clinical digital infrared thermal imaging. BioMedical Engineering OnLine.

[B20] Rose AD, Kanade V (2006). Thermal imaging study comparing phacoemulsification with the sovereign with WhiteStar System to the legacy with AdvanTec and NeoSoniX System. American Journal of Ophthalmology.

[B21] Murthy R, Pavlidis I (2006). Non-contact measurement of breathing function. IEEE Engineering in Medicine and Biology Magazine.

[B22] Morimoto T, Takada K, Huiya H, Yasuda Y, Sakuda M (1991). Changes in facial skin temperature associated with chewing efforts in man: a thermographic evaluation. Archs oral Biol.

[B23] Tu J, Tao H, Huang T (2007). Face as mouse through visual face tracking. Computer Vision and Image Understanding.

[B24] Steketee J (1973). Spectral emissivity of the skin and pericardium. Phys Med Biol.

[B25] L-3 Communications Infrared Products, Thermal-Eye 2000B/300D. http://gotoinfrared.com/L3.htm.

[B26] Zaproudina N, Varmavuo V, Airaksinen O, Narhi M (2008). Reproducibility of infrared thermography measurements in healthy individuals. Physiological Measurement.

[B27] Otsu N (1979). A Threshold Selection Method from Gray-Level Histograms. IEEE Transactions on Systems, Man, and Cybernetics.

[B28] Gonzalez RC, Woods RE, Eddins SL (2004). Digital Image Processing Using MATLAB.

[B29] Barron JL, Fleet DJ, Beauchemin SS, Burkitt TA (1996). Performance of optical flow techniques. Proceedings of IEEE Computer Society Conference on Computer Vision and Patter Recognition (CVPR): 1992; Los Alamitos, CA.

[B30] Uematsu S (1986). Symmetry of skin temperature comparing one side of the body to the other. Thermology.

[B31] Bailar JC, Meyer EA, Pool R, Editors (2007). Assessment of the NIOSH head-and-face anthropometric survey of US respirator users.

[B32] DeCarlo D, Metaxas D, Stone M (1998). An anthropometric face model using variational techniques. Proceedings SIGGRAPH; 1998; Orlando, FL.

[B33] Moriyamashi T, Tagucihi H, Mishima Y (1996). Relation between the brain waves, face temperature and blood pressure using nonintrusive blood pressure monitor and the environments. Proceedings of the 35th SICE Annual Conference International Session Papers: July 24–26 1996; Tottori, Japan.

[B34] (2005). MobIR® M4 Thermal Camera User Manual.

[B35] Wang J, Sung E (2007). Facial feature extraction in an infrared image by proxy with a visible face image. IEEE Transactions on Instrumentation and Measurement.

